# *Tetratricopeptide Repeat 2* Is a Quantitative Trait Locus That Controls Seed Size

**DOI:** 10.3390/ijms26178310

**Published:** 2025-08-27

**Authors:** Zhuolun Wang, Stephanie Cara, Seung Y. Rhee, Bernard A. Hauser

**Affiliations:** 1 Department of Biology, University of Florida, Gainesville, FL 32611, USA; doro555hy@gmail.com (Z.W.); cara.s@ufl.edu (S.C.); 2 Plant Resilience Institute, Departments of Biochemistry and Molecular Biology, Plant Biology, and Plant, Soil, and Microbial Sciences, Michigan State University, East Lansing, MI 48824, USA; rheeseu6@msu.edu; 3 Plant Molecular and Cellular Biology Program, University of Florida, Gainesville, FL 32611, USA

**Keywords:** *Arabidopsis thaliana*, assimilate transfer, QTL, seed development, seed size

## Abstract

Seed size is a key trait affecting evolution and agronomic performance by influencing seedling establishment in natural populations and crop yields. The *Arabidopsis thaliana* Seed Size QTL1 (SSQ1) locus explains 10–15% of the variation in seed size. We report here that the causal gene for this locus is *Tetratricopeptide Repeat Protein 2* (*TPR2*), which encodes a co-chaperone. Expressing *TPR2* across ecotypes and genotypes showed consistent dosage effects. Each additional *TPR2*^Col-0^ allele increased seed mass and volume by 10–14% with high reliability in Col-0, Sha, Tsu-1, and *tsu2* genetic backgrounds. Reciprocal genetic crosses indicated that this locus acts maternally, consistent with female sporophytic or female gametophytic mutations. To elucidate how *TPR2* regulates seed size, the biomass composition of seeds was measured. While oil content remained unchanged, sucrose levels were markedly elevated in *TPR2*^Col-0^ transformant lines and reduced in *tpr2* mutants. Interestingly, heterologous expression of *TPR2*^Col-0^ across genetic backgrounds increased seed protein accumulation by 18% on average. Based on these changes in sucrose and protein levels, potential modes of action for *TPR2* are discussed.

## 1. Introduction

Seeds are the main plant products for human and animal consumption, as well as a primary source for biofuels. Seed size, a critical factor controlling crop yields, is a quantitative trait regulated by the interplay of genetic, environmental, and hormonal factors. While genetic determinants establish the baseline for seed size variation, environmental influences, such as soil nutrient and water availability, temperature fluctuations, and light conditions, dynamically modulate seed development and size by altering maternal resource allocation and transcriptional programs within the embryo sac [1]. Plant hormones, including auxins (regulating cell division patterns in the endosperm), gibberellins (promoting cotyledon expansion), and cytokinins (delaying seed coat lignification), control seed development and fine-tune seed growth [2]. Hormones not only regulate the rate of cell division but also integrate resource allocation into seeds with the physiological state of the plant and its environmental conditions [3,4,5,6,7,8]. Understanding how hormone physiology and environmental cues interact with the genetic architecture regulating seed size enhances our understanding of seed evolution and development, and enables the formulation of crop improvement strategies [2].

During seed development, seed size is determined through regulation of three regions of the ovule: the maternal tissue, which sets the upper limit for seed growth; the endosperm, which controls nutrient uptake into the embryo sac; and the embryo, which determines the demand for nutrients through embryogenesis and storage reserve synthesis [9,10,11]. During early development, cell divisions in the maternal integument, endosperm, and embryo establish potential for the seed size [11,12]. This phase relies on auxin concentration in the chalaza to drive integument cell proliferation [8] and transcriptional networks polarizing the embryonic axis [2]. Subsequently, during seed filling, maternal nutrients are transported through the endothelium to the embryo sac, where invertases and expansins mediate cotyledon cell expansion [11]. Environmental stresses disrupt this process by suppressing sucrose transport into the embryo sac [13,14], thereby preventing the realization of size potential. Ultimately, seed size reflects a tripartite regulation: the maternal integument’s physical constraints, the endosperm’s nutrient transport efficiency, and the embryo’s demand-driven storage expansion. Mutant analyses have shown that decreasing the number of cell divisions in the endosperm, integuments, or embryo reduces seed size [15,16,17,18,19,20]. Furthermore, crosstalk between the endosperm and maternal tissues modulates seed size [21].

Despite the knowledge gained from these genetic factors that affect seed size, we do not yet understand the molecular mechanisms by which plants modulate seed size in response to environmental changes. Genetic loci regulating seed size were identified in many quantitative trait locus (QTL) mapping studies and genome-wide association studies (GWASs) [22,23,24,25]. In *Arabidopsis*, QTL regions reported by Alonso-Blanco et al. [26] were corroborated by Guo et al. [22], using Sha (~18 µg/seed) and Tsu-1 (~28 µg/seed) Arabidopsis ecotypes; they identified 12 Seed Size QTLs (SSQs). The Tsu-1 accession was collected from Tsushima Island, which is located in the Korean Strait near Kyushu, Japan. The Sha accession comes from Shahdara Mountains in Tajikistan (elevation ~3400 m), where temperatures are substantially colder. When grown under identical laboratory conditions, Tsu-1 plants are bigger than Sha and Col-0 plants. In addition, Tsu-1 seeds from the temperate Japanese accession are up to twice the size and mass of Sha from the arid Asian mountains. For this reason, these quantitative trait genes (QTGs) likely include alleles involved in environmental adaptation. Despite the wide use of these methods in numerous plant species, only two genetic variants regulating seed size have been found from QTL or GWAS studies [27,28]. This gap highlights the urgent need for advances in QTG identification [29].

To identify causal genes underlying seed size variation, we sought to identify the causal gene underlying SSQ1, a large-effect QTL that was estimated to account for 10–15% of the seed size variation [26]. We evaluated the size of all available knock-out mutant seeds within SSQ1 and identified a gene, *Tetratricopeptide Repeat Protein 2 (TPR2)*, which causes a 16% change in seed size when transformed into the wild-type (Col-0) genome. The cloning of causal genes underlying seed size variation, as described in this study, bridges fundamental insights in plant development with translational strategies for engineering climate-resilient cultivars.

## 2. Results

### 2.1. Mutations Altering Seed Size in the SSQ1 Interval

Size can describe a variety of distinct physical properties: length, area, volume, cross-sectional area, mass, or weight. In this work, seed size was measured in one of two ways: seed mass and estimated seed volume. Seed volume was calculated from length and width measurements. We assumed seeds were prolate spheroids, and the volume was estimated as follows:

$$V=\frac{4}{3}\pi {\left(\frac{W}{2}\right)}^{2}\left(\frac{L}{2}\right)=\frac{\pi W^{2}L}{6}$$

where *V* = volume, *W* = width, and *L* = length of the seed.

Seed mass was directly measured using a microscale. If seed density is the same between genotypes, then seed mass can be used to accurately predict seed volume and vice versa. Both seed size and seed volume were measured for all three accessions; we found that these two variables showed excellent correlation (cov = 0.98). This tight relationship validates the interchangeable use of seed weight and seed volume in our analyses of these genotypes and accessions.

**Figure 1:**
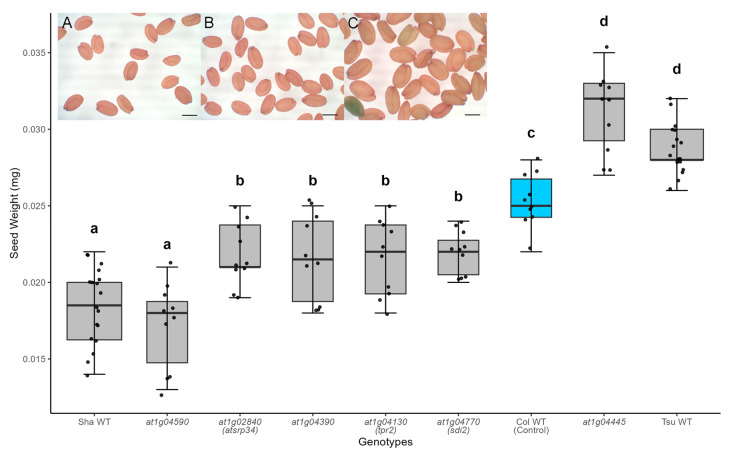
Six mutants within the SSQ1 interval exhibited significantly altered seed mass (N ≥ 10). Seeds produced by the At1g04445 mutant were substantially larger (*p*-value << 0.05). Seeds from the other five mutants were smaller than Col-0 wild-type controls (blue box). This change in seed mass was found in multiple generations. Significant differences were calculated using one-way ANOVA and Tukey HSD and denoted by letters. Inset: Micrographs of (**A**) Sha, (**B**) Col-0, and (**C**) Tsu-1 seeds are shown (bar = 1 mm). This experiment was repeated twice, and a representative dataset was selected for this figure. Parameters for this box-whisker plot are described in the methods.

An *Arabidopsis* seed size QTL named Seed Size QTL1 (SSQ1) was previously reported to drive 10–15% of the variation in seed size [22]. SSQ1 was identified from QTL mapping between Sha x Tsu-1 F2 populations. The inset of Figure 1 shows Sha, Col-0, and Tsu-1 accessions, respectively. The SSQ1 locus lies on chromosome 1 between 6.5 cm and 15.3 cm, harboring 603 genes [22]. To identify the causal gene in this interval, we characterized T-DNA insertion mutant lines in the Col-0 background for 522 of the 603 genes in this interval. T-DNA inserts were not available for 81 genes in the interval. Using the SeedSize tool [30], we observed that 43 mutants significantly differed in size from control Col-0 seeds (Welch’s *t*-test, *p*-value < 0.05, *n* ≥ 30, Supplementary Figure S1).

Since these mutants were propagated at different times, we posited that environmental variability likely caused some of these differences in size. To determine which of these mutants exhibited heritable changes in seed size and reduce the influence of environmental variation, these 43 mutant lines were propagated simultaneously in a single tray. Five mutants had significantly smaller seeds, and one had significantly larger seeds than Col-0 (Figure 1).

SSQ1 has previously been reported to contribute 10% to 15% of the seed size change [26]. Four mutants exhibited ~10% decrease in seed size: Salk_073054 (At1g04130), Salk_088004 (At1g04390), Salk_010894 (At1g02840), and Salk_091618 (At1g04770) (Figure 1). Two mutants had ~30% changes in seed size: Salk_079219 (At1g04590) (decrease) and Salk_130261 (At1g04445) (increase).

### 2.2. A Cosmid with the TPR2^Col-0^ Transgene Increases Seed Size

**Figure 2:**
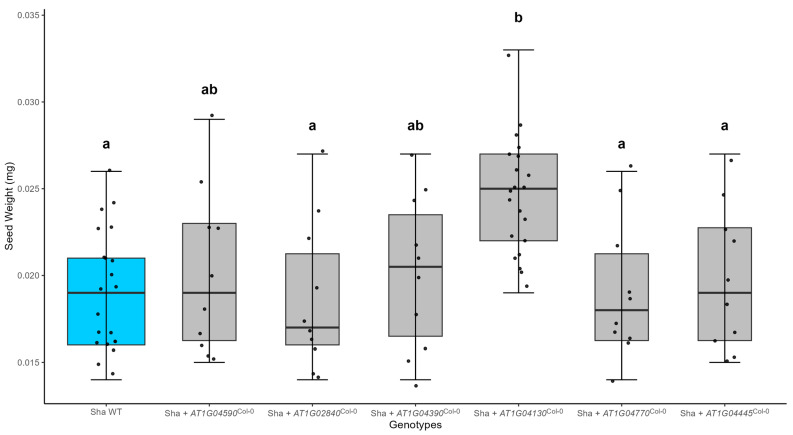
Potential SSQ1 genes were screened by transformation of cosmids containing the gene of interest. Seed mass of cosmid transformants in the Sha genetic background (N ≥ 10). For five genes, seed size did not differ from the Sha control plants (blue box). The *At1g04130* (*TPR2*^Col-0^) transgene significantly increased seed mass in the Sha accession. Significant differences were calculated using one-way ANOVA and Tukey HSD and denoted by letters. Dots represent seeds counted from each genotype. Parameters for this box-whisker plot are described in the methods.

To identify the causal gene from the six candidates, we sought to identify the gene that changed seed size in Sha and/or Tsu-1. We hypothesized that if a causal gene contributed to seed size, there should be a change in seed size that is proportional to the number of transgenic inserts at least one of the parental backgrounds. To test this hypothesis, we obtained Bacterial Artificial Chromosome (BAC) clones from Col-0, which contained the six candidate genes in the SSQ1 interval. Cosmid libraries were made from the BAC clones by cloning partially digested *Sau*3A1 fragments (20–25 kb) into pOCA28. Cosmids containing the six genes were identified and transformed into Tsu-1, Col-0, and Sha plants. In the Sha background, *At1g04130* (*TPR2*) transgenic lines increased seed mass by 34.6% (ANOVA, *p*-value = 10^−7^). Seed mass was not altered in cosmid transformants containing *At1g04770* (*SDI2*), *At1g04590*, *At1g02840* (*SRP34*), *At1g04445*, or *At1g040390* transgenes (Figure 2). The *TPR2^Col-0^* transgene significantly increased seed size in both Col-0 (11.8%) (Supplementary Figure S1) and Sha (34.6%) genetic backgrounds (Figure 2).

### 2.3. TPR2 Acts Maternally to Regulate Seed Size

**Figure 3:**
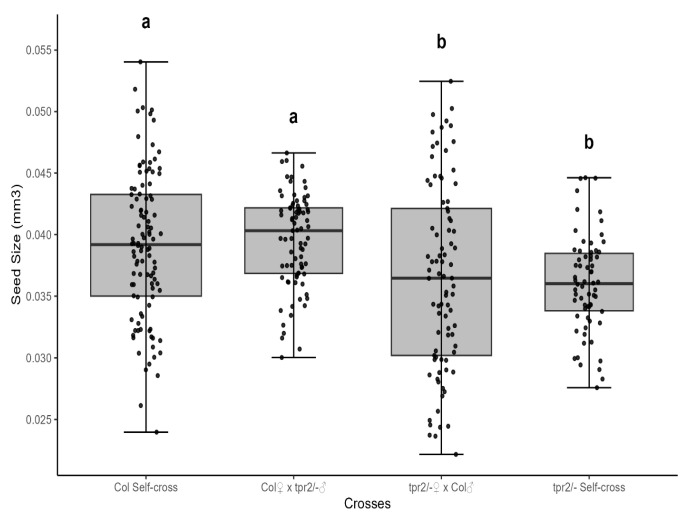
Reciprocal crosses of *tpr2* mutants with wild-type plants show that this locus acts maternally (*n* = 109). When using wild-type (Col-0) female parents in a cross, seed size was significantly bigger than when *tpr2* mutants were used as the female parent. Conversely, changing the genotype of the male parent did not significantly alter seed size in the F_1_ seeds. These data are consistent with female sporophytic or female gametophyte mutations. For each sample, seeds were derived from 10 different sets of parents. Statistical significance was determined by one-way ANOVA with Tukey’s post hoc test. Significant differences are denoted by letters. Parameters for this box-whisker plot are described in the methods.

To determine if *TPR2* alleles affect the chalaza, integuments, embryo, or gametophyte cells in an ovule, we analyzed seed size from reciprocal crosses between *tpr2* mutants and wild-type (Col-0) plants. Altered seed mass was observed only when maternal plants were homozygous for the *tpr2* allele (Figure 3). When *tpr2* female plants were crossed to Col-0, seed mass of the F_1_ seeds was significantly reduced (Figure 3). In contrast, maternal Col-0 plants produced F_1_ seeds with wild-type seed mass regardless of the genotype of the pollen donor; these seeds were significantly larger than those generated by maternal *tpr2* mutants (Figure 3). These results indicate that *TPR2* is maternally inherited, so the primary TPR2 function occurs in integument, chalaza, or female gametophyte tissues to regulate seed mass.

### 2.4. TPR2 Increases Seed Size and Mass in All Accessions

**Figure 4:**
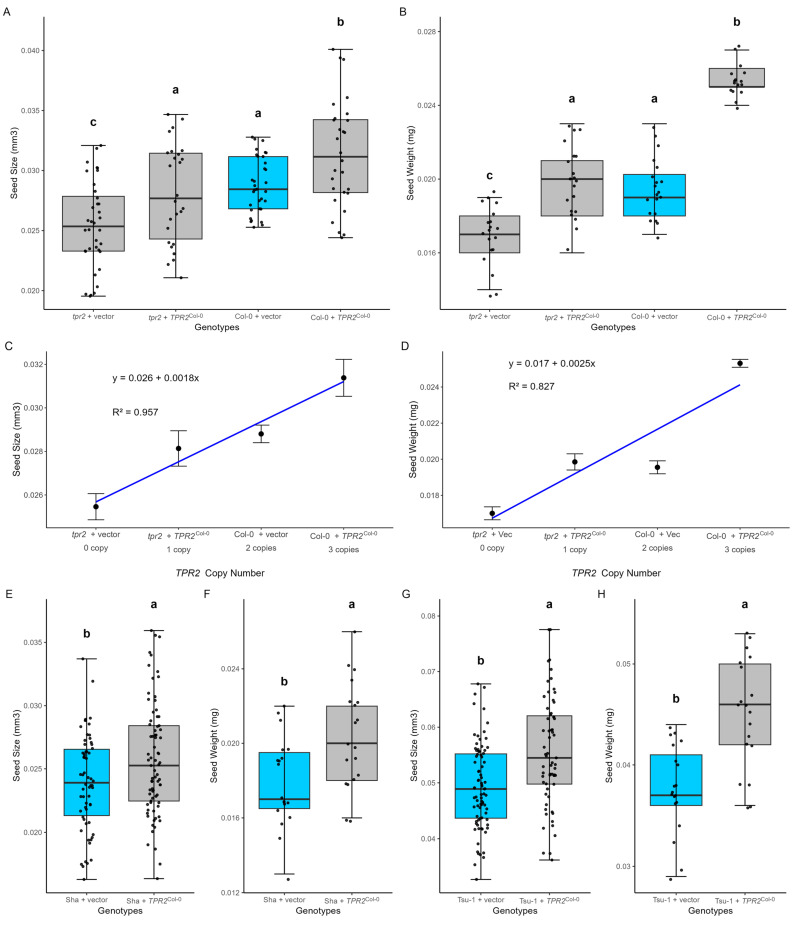
Seed mass of different genotypes: Tsu-1, Sha, Col-0, and *tpr2* mutants, as well as these genotypes transformed with (+) a *TPR2^Col-0^* allele or the vector lacking a transgene (vector). (**A**,**B**) In the Col-0 background, the *TPR2^Col-0^* transgene significantly increased seed mass and complemented the phenotype of *tpr2* mutants. When compared to the wild-type seeds, *tpr2* knockout lines showed decreased seed mass. The effects of increasing the *TPR2^Col-0^* dosage are plotted by (**C**) seed volume and (**D**) seed mass. As indicated by the R^2^ values, most of the change in seed size can be attributed to the number of *TPR2^Col-0^* alleles. (**E**,**F**) In the Sha background, the *TPR2*^Col-0^ transgene generated larger seeds than controls. (**G**,**H**) Expression of *TPR2^Col-0^* in the Tsu-1 accession increased seed mass by 12.6%. Statistical significance was determined by one-way ANOVA with Tukey’s post hoc test. Significant differences are denoted by letters. Analyzed T_2_ seeds were the progeny of T_1_ plants with a single transgene. This experiment was repeated four times, and a representative dataset was selected for this figure. Parameters for these box-whisker plots are described in the methods. The empty vector without a transgene inserted (vector) is a control (blue box).

Given that cosmids contain DNA inserts of 20–25 kb in length, the transformed cosmid insert contained not only the gene of interest but also other DNA fragments, including nearby genes and non-coding regions. To exclude the influence of these additional DNA elements, we transformed a single-gene construct containing only *TPR2*^Col-0^ into Col-0, Sha, and Tsu-1 plants, and measured seed mass (Figure 4). *TPR2*^Col-0^ *(At1g04130)* increased both seed size and seed mass in T-DNA insertion mutant lines and all three accessions (Figure 4). Transgenic expression of *TPR2*^Col-0^ in Sha and Tsu-1 increased seed size by 7.4% (*p*-value = 0.01) and 12.6% (*p*-value = 10^−4^), respectively (Figure 4E–H). These results indicated that expression of the *TPR2*^Col-0^ transgene increased seed size and mass by approximately 10% in multiple genetic backgrounds, which was consistent with the mutant analysis and cosmid transformation data. Furthermore, the addition of the *TPR2*^Col-0^ into the *tpr2* mutant (Col-0 background complemented the small seed phenotype (Figure 4A,D). These results are consistent with *TPR2* being a causal gene affecting seed size within the SSQ1 locus.

### 2.5. Additional TPR2^Col-0^ Alleles Add ~10% to Seed Size

An allelic series for *TPR2*^Col-0^ was created with transgenic, wild-type, and mutant plants. All analyzed seeds were T_2_ progeny of T_1_ plants with a single transgene. All T_1_ transformants with a single *TPR2*^Col-0^ insert would be hemizygous for the transgene and contain two endogenous *TPR2* alleles in maternal portions of developing T_2_ seeds. To determine if a single transgene was present in each transformant, the ratio of Basta resistant and sensitive T_2_ plants was evaluated for each T_1_ transformant (see Section 4.3). Diploid Col-0 plants had two *TPR2* alleles. Col-0 plants with a *TPR2*^Col-0^ transgene had three alleles. The *tpr2* mutant has no functional alleles. Complementation of this *tpr2* mutant with the *TPR2*^Col-0^ transgene results in a single functional *TPR2*^Col-0^ allele. When seed size is plotted as a function of the number of functional *TPR*^Col-0^ alleles, each additional allele increased seed mass by an average of 2.5 µg (Figure 4D). In addition, regression analysis showed that copy number accounts for more than 80% of the change in seed size (Figure 4C,D). This analysis shows each *TPR2*^Col-0^ allele adds 10–14% to seed size.

Transformation of the *TPR2*^Col-0^ allele into the Sha accession increases seed size, indicating that *TPR2^Sha^* allele is not dominant over the *TPR2*^Col-0^ allele. So, the small seed phenotype in Sha accessions is not caused by a dominant deleterious allele or dominant repressor. Removing dominant classes of mutations from Muller’s morphogenic classes leaves amorphs and hypomorphs, categories with reduced gene activity. Since the *TPR2*^Col-0^ allele contributes to seed size, we hypothesize that the *TPR2^Sha^* allele functions similarly but less efficiently.

### 2.6. TPR2 Transcript Abundance Similar Among Arabidopsis Accessions

Quantitative PCR was used to measure the transcript abundance in the Arabidopsis accessions to determine if mRNA abundance explained the observed seed phenotype. This gene is expressed at peak levels in dry seeds and developing ovules, especially in the chalaza, integuments, and endosperm adjacent to the chalaza [31]. RNA was isolated from flowers at anthesis since *TPR2* transcripts are highest in ovules at this time [31]. Two transcription start sites are reported for *TPR2*. The shorter transcript will not encode two of the three tetratricopeptide repeats. Primers annealed at the 3′ end of the gene, which measures both of these transcripts. Since expression was highest in the Col-0 accession, we normalized *TPR2* transcript levels relative to this sample. In the three replicates, the related transcript abundance was 0.91 ± 0.14, 1.00 ± 0.16, and 0.98 ± 0.08 for the Sha, Col-0, and Tsu-1 accessions, respectively. These mRNA levels were not significantly different by one-way ANOVA analysis (*p* < 0.05).

### 2.7. RNA Variants Among TPR2 Alleles

**Figure 5:**
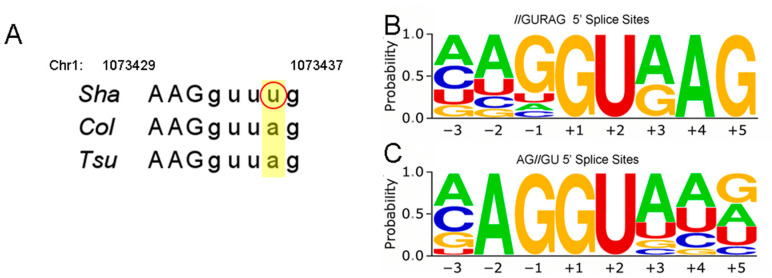
Genetic variation at the third intron/exon boundary for the *TPR2* gene. (**A**) Sequence alignment of Sha, Col-0, and Tsu-1 alleles reveals an SNP that occurs near a 5′ splice site. The Sha sequence contains a transversion (circled in red). Recent work on 5′ splice recognition sequences reveals that there are two consensus sequences: (**B**) //GURAG 5′ (**C**) and AG//GU. The height of the letters corresponds to nucleotide frequencies at positions −3 to +5.

To identify the genetic variation in *TPR2* alleles, we examined *TPR2* sequences from accessions of interest using the 1001 genomes database [32]. This database reveals that the Cvi accession has a missense mutation at position 1073941. The *TPR2* gene sequence is identical between Col-0 and Tsu-1, but there is an SNP at position 1074135 in the Sha accession, which alters the 5′ splice recognition sequence of the third exon. The consensus 5′ splice site recognition sequence is (A/C)AGgu(a/g)ag, where exon nucleotides are uppercase and intron nucleotides are lowercase. The Sha accession differs at the third and fourth nucleotide in the intron (shaded), while the Col-0 and Tsu-1 accessions differ only at the third (a/g) nucleotide in the intron (Figure 5). This altered splice recognition site in the Sha accession could affect the activity of the *TPR2* gene. We complemented the Sha accession with an allele from the Col-0 genome (Figure 2 and Figure 4E,F). The transformation experiments indicated that the *TPR2*^Col-0^ allele increased seed mass and seed size in all three genetic backgrounds (Figure 4).

### 2.8. TPR2^Col-0^ Activity Affects Sucrose and Protein Levels in Seeds

**Figure 6:**
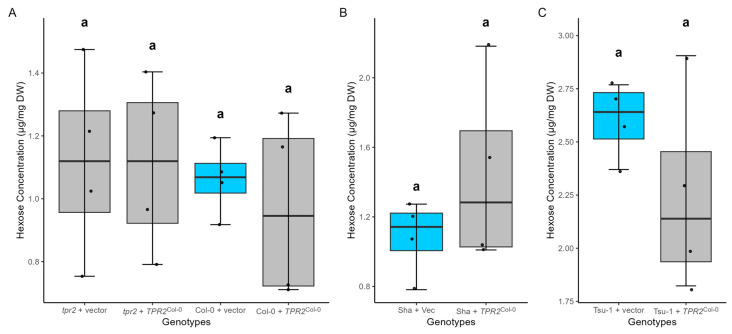

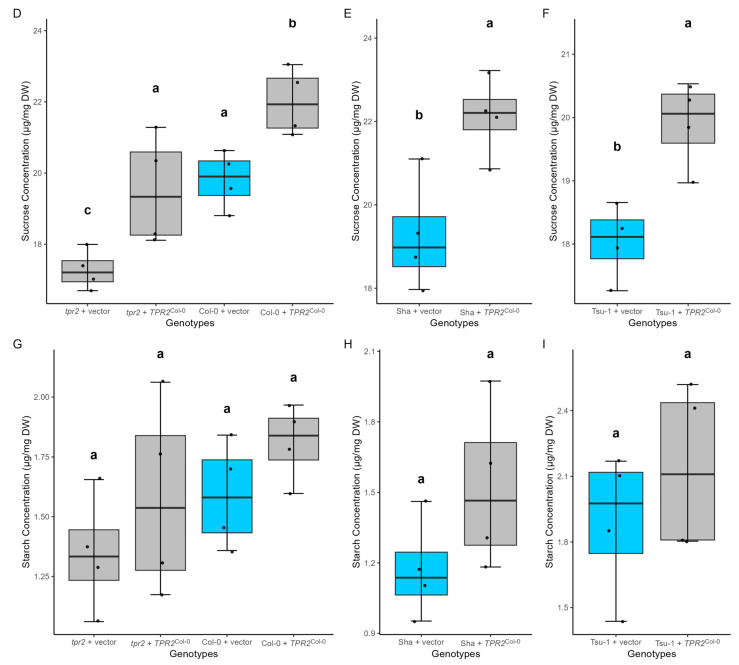
Carbohydrate concentration on a seed dry mass basis (*n* = 4 biological replicates). (**A**–**C**) Concentration of hexose (glucose + fructose) in Col-0, Sha, and Tsu-1 accessions. (**D**–**F**) Concentration of sucrose in Col-0, Sha, and Tsu-1. (**G**–**I**) Concentration of starch in Col-0, Sha, and Tsu-1. Data were the mean of four independent measurements of T_2_ seeds from four individual T_1_ plants. The vector without a transgene inserted (vector) is a control (blue box). Analyzed T_2_ seeds were the progeny of T_1_ plants with a single transgene. Significant differences were calculated using one-way ANOVA and Tukey HSD and denoted by letters. Parameters for these box-whisker plots are described in the methods.

**Figure 7:**
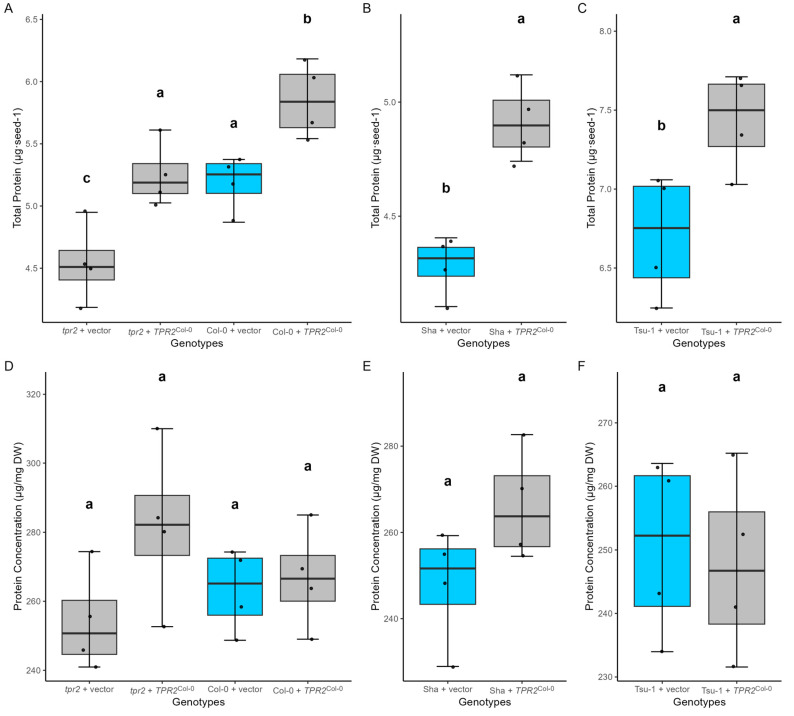
Protein contents in different genotypes (*n* = 4 biological replicates). (**A**–**C**) The total amount of protein per seed in Col-0, Sha, Tsu-1, and their transgenic lines. (**D**–**F**) Protein concentration on the seed dry mass basis in Col-0, Sha, Tsu-1, and their transgenic lines. Analyzed T_2_ seeds were the progeny of T_1_ plants with a single transgene. The empty vector without a transgene inserted (vector) is a control (blue box). Data were the mean of four independent measurements of T_2_ seeds from four individual T_1_ plants. Significant differences were calculated using ANOVA and Tukey HSD and denoted by letters. Parameters for these box-whisker plots are described in the methods.

To understand the nature of the seed size differences among the genotypes in this study, we measured soluble carbohydrate and starch content in the seed. In *tpr2* seeds, less sucrose accumulated relative to the seeds from plants containing 1–3 copies of the *TPR2*^Col-0^ allele (Figure 6D and Supplementary Table S1). In contrast, presence of the *TPR2*^Col-0^ transgene substantially increased sucrose content in all three genetic backgrounds (Figure 6D–F). Seeds from *TPR2*^Col-0^ transgenic Sha and Tsu-1 lines accumulated 24% and 20% higher levels of sucrose, respectively (Supplementary Figure S2). In *tpr2* seeds, we did not observe statistically significant changes in glucose, fructose, or starch levels (Figure 6). In addition, 13% less protein accumulated in *tpr2* mutants than in Col-0 seeds (Figure 7A). On a per-seed basis, expression of *TPR2*^Col-0^ affected the amount of protein per seed; Sha, Col-0, and Tsu-1 accessions averaged 4.9, 5.9, and 7.4 μg protein/seed, respectively (Figure 7A–C). In Sha plants transformed with a *TPR2*^Col-0^ transgene, 15% more protein was stored in seeds than in the Sha controls (Figure 7B). *TPR2*^Col-0^ transgene expression in Col-0 and Tsu-1 similarly elevated protein accumulation by 13% and 11%, respectively (Figure 7A,C).

**Figure 8:**
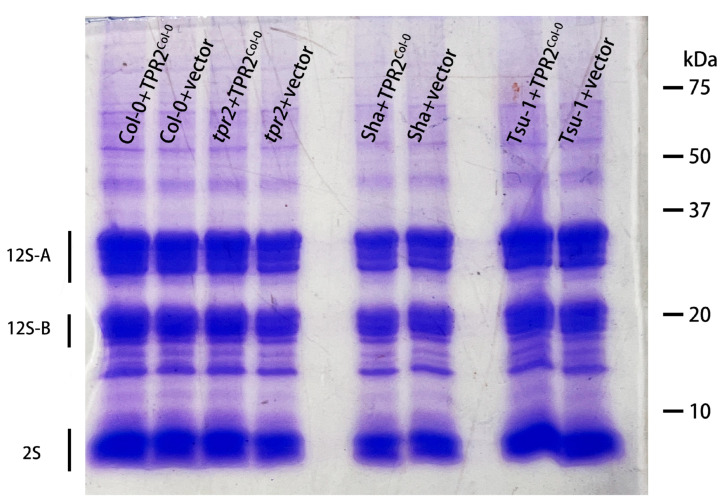
SDS-PAGE of proteins from dry seeds of different genotypes. After Coomassie blue staining, seed storage proteins (SSPs) predominate; acidic (12S-A) and basic (12S-B) subunits of 12S globulin (cruciferin) and 2S albumin (napin) are labeled. The vector without a transgene inserted (vector) is a control. A total of 20 μg of protein was loaded in each lane.

To determine if TPR2 activity alters seed storage proteins (SSPs), proteins were extracted from dry seed and analyzed by gel electrophoresis. Regardless of the genotype, there were no gross changes in the relative amounts of SSPs; the intensity of 2S albumin (napin) appears unchanged in different *TPR2* genotypes; however, the intensity of the 12S globulins appears fainter in the Sha accession (Figure 8).

**Figure 9:**
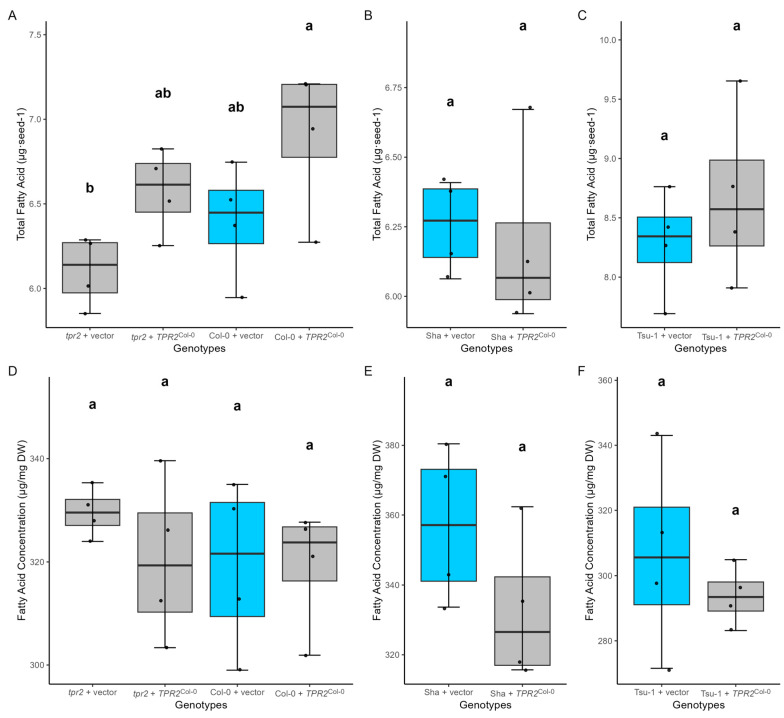
Fatty acid levels in different genotypes (*n* = 4 biological replicates). (**A**–**C**) Total amount of fatty acid levels per seed in Col-0, Sha, Tsu-1, and their transgenic lines. (**D**–**F**) Fatty acid concentration on the seed dry mass basis in Col, Sha, Tsu-1, and their transgenic lines. The empty vector without a transgene inserted (vector) is a control (blue box). Data were the mean of four independent measurements of T_2_ seeds from four individual T_1_ plants. Significant differences were calculated using one-way ANOVA and Tukey HSD and denoted by letters. Parameters for these box-whisker plots are described in the methods.

When comparing oil accumulation in seeds, we did not find a significant variation in lipid content among genotypes (Figure 9), indicating that TPR2 does not impact oil accumulation. These data show that the *TPR2*^Col-0^ transgene increases protein and sucrose accumulation during seed formation.

## 3. Discussion

### 3.1. A Strategy to Find QTG Altering Seed Mass

We developed a strategy to identify the genes controlling seed size. Once the boundaries of a QTL are delineated, all of the homozygous mutants in this interval were imaged and the dimensions measured with the SeedSize program. For mutants with significant changes in seed dimensions, we checked that this change in size was heritable and not an environmental effect by growing mutants and controls under the same growth conditions for another generation. BAC DNA containing a locus of interest was cloned into a cosmid at random and then screened by PCR for the locus. Cosmid transformants containing the gene of interest was then be evaluated to determine if the transgene affected the trait of interest. This was then be followed by single-gene transformants to determine if the phenotype was complemented by the transgene. These steps were used to identify the *TPR2* locus as a causal gene regulating seed size in the SSQ1 QTL.

### 3.2. TPR2 Splice Variants

*TPR2* encodes a number of transcript variants; two variants involve exon3 (Supplementary Figure S3). If one of these RNA variants requires the NN/gurag splice recognition sequence [33] for exon 3, this sequence is altered in the *TPR2*^Sha^ allele (Figure 5), which might lead to altered activity. Altered frequency of the use of transcription start sites or splice variants could explain this phenotypic difference between Sha and Col-0 alleles.

### 3.3. TPR2 Effects on Sucrose and Protein Accumulation in Seeds Is Consistent with a Seed Size Phenotype

*TPR2* encodes a carboxylate clamp (CC)–tetratricopeptide repeat (TPR) protein [34]. TPR domains contain two α-helices oriented in an anti-parallel manner, which act as a scaffold to recruit partner proteins.

In the introduction, three mechanisms that are reported to lead to increased seed size are described: increased mobilization of resources from the endothelium into the embryo sac, activity of hormones involved in seed development, and altered proliferation of cells in the ovule. Based on the inheritance of *TPR2* and its effect on sucrose and protein accumulation in the seed, we hypothesize that *TPR2* function in the chalaza, integuments or female gametophyte to regulate nutrient partitioning during seed development.

### 3.4. TPR2 Affects Sucrose Levels in Seeds

*TPR2*^Col-0^ alleles affect sucrose accumulation in seeds when expressed per unit dry weight (Figure 6) and per seed (Supplementary Figures S1 and S4). These measurements might reflect sucrose levels earlier in seed development during assimilate transfer. High sucrose levels fuel more rapid endosperm cell growth and division, resulting in increased photo-assimilate uptake. These resources can later be used for protein and fatty acid biosynthesis. Researchers hypothesize that the hexose/sucrose ratio affects many metabolic activities during seed development [35,36,37]. A high hexose/sucrose ratio correlates with rapid cell division during the early morphogenesis phase of seed development, while a low hexose/sucrose ratio corresponds to greater seed filling and accumulation of storage resources [37,38]. The ratio of hexose/sucrose levels differed in dry seeds (Figure 6); however, it remains to be determined if this was also the case during seed filling. TPR2 could facilitate the establishment of protein complexes in endothelial cells with efficient transfer properties to promote resource allocation into seeds. While long-standing hypotheses predict that increased transport of sucrose across the endothelium will lead to greater anabolic metabolism, the data described here are insufficient to infer a causal relationship between elevated sucrose levels and larger seeds. Therefore, the relationship between *TPR2* alleles and sugar metabolism should be investigated further during seed development.

### 3.5. TPR2 Proteins and Hormone Activity During Seed Development

Proteins containing tetratricopeptide repeat motifs have been reported to act during plant hormone responses, including abscisic acid (ABA), ethylene, and gibberellin (GA). Among these, ABA plays a central role in regulating seed development and maturation. Rosado et al. [39] identified *TTL1*, an *Arabidopsis* TPR-containing protein, as a positive regulator of ABA signaling during seed development and germination under osmotic stress. The expression of some dehydration-responsive genes in seeds was reduced in *ttl* mutants. Notably, TPR2 interacts with ABI5, a core ABA-responsive transcription factor [40], suggesting a shared regulatory axis in ABA signaling during seed development. Furthermore, both TTL1 and TPR2 interact with Hsp90/Hsp70 [34]. This raises the possibility that TPR2, like TTL1, may stabilize ABA signaling components (e.g., ABI5) or modulate stress-responsive pathways, thereby influencing seed development.

While ABA governs stress adaptation and seed development, ethylene signaling modulates reproductive organ development and yield-related traits. For instance, TRP1 (another TPR-containing protein) physically interacts with the ethylene receptor ERS1 and regulates development in *Arabidopsis*. Ectopic overexpression of TRP1 in wild-type *Arabidopsis* induced ethylene-associated pleiotropic phenotypes, including reduced fertility, aberrant silique development, and significant reductions in seed yield [41]. Analogous developmental defects were observed in transgenic tomato lines overexpressing *SlTPR1*, the ortholog of *TPR1*, demonstrating evolutionary conservation of this regulatory mechanism [42].

TPR domain-containing proteins also affect seed development in a GA-dependent manner, which is critical for balancing seed growth and dormancy. *SPINDLY (SPY)* encodes the N-terminal portion of a TPR domain and is a negative regulator of GA response [43,44]. Overexpression of *SPY* increased seed dormancy [45]. After fertilization, ovules synthesize GA, which promotes fruit growth and development [46].

### 3.6. TPR2-Mediated Effects on Cell Growth

TPR-containing proteins were first found to promote the cell cycle [47,48]. During the early morphogenesis of seed structures, integument cells undergo rapid cell division and cell expansion to create a large seed cavity, which is a crucial determinant of sink strength [49]. The integuments regulate carbon supply from the maternal tissue [11]. In addition, the vascular trace terminates at the chalaza and acts as a valve to modulate the amount of carbon input [50]. Thus, the maternal chalaza regulates carbon flux from source to sink. *TPR2* mRNA levels are highest in the chalaza and integuments during seed filling [31]. These data lend support to the hypothesis that *TPR2* promotes the growth of integument and chalaza cells, thereby promoting assimilate movement into developing seeds.

## 4. Materials and Methods

### 4.1. Plant Growth Conditions and Seed Measurement

*Arabidopsis thaliana* plants were grown as previously described [51]. Temperature was maintained between 20 and 22 °C, relative humidity set at 60%, continuous illumination at 100 μmol photons m^−2^ s^−1^, and plants watered every second day as required. With the exception of cosmid transformants, all seed size data presented in the figures were conducted in multiple independent experiments. During the course of these experiments, Arabidopsis seed size showed significant environmental plasticity. We believe that the most important environmental parameter is soil moisture content, so efforts were made to keep this as constant as possible by checking the weight of pots before and after watering. The data in the figures are from a single experiment because the environmental effects and interaction effects usually varied among independent experiments, though the trends among genotypes were consistent across experiments and ANOVA classes (denoted by a, b, c, etc.) remained consistent, demonstrating reproducibility in genotypic effects. T-DNA insertion mutants of the 603 genes from the SSQ1 QTL region (6.5–15.3 cm on Chr1) were obtained and characterized from ABRC stocks (Ohio State University). Seed sizes were analyzed by ImageJ version1.44, and SeedSize software was used between 4/13 and 6/18 [31]. Masses of individual seeds were measured using a Mettler Toledo MT5 microbalance. Mutants with significantly altered seed size were propagated together to ensure the change in size was heritable and not caused by environmental variation. T-DNA insertion of Salk_073054 (*At1g04130*; *TPR2*) and Salk_091618 (*At1g04770*; *SDI2*) mutants were confirmed by Polymerase Chain Reaction (PCR) using primers listed in Supplementary Table S2. Two gene-specific primers from upstream and downstream of coding sequences were used to verify the presence of wild-type alleles. One gene-specific primer and one primer from the T-DNA border sequence were used to confirm the presence of the mutant allele.

### 4.2. Cosmid and Single-Gene Cloning

Bacterial Artificial Chromosome (BAC) T1G11 was obtained from ABRC and partially digested by Sau3AI. DNA was fractionated on a 10–40% sucrose gradient by centrifugation at 25,000 rpm for 16 h in SW-40 rotor [52]. DNA fragments ranging from 15- to 25-kb were inserted into the *Bam*HI site of pOCA28 [53]. Cosmids were packaged using GigaPack, as recommended by the manufacturer (Stratagene, La Jolla, CA, USA), and propagated in *E. coli* strain JM109. Constructs containing the gene of interest were identified by colony lift hybridization using digoxigenin-labeled probes. The ends of positive cosmids were sequenced to confirm the existence of the target gene and its entire promoter region. Cosmids were introduced into *Agrobacterium tumefaciens* strain ASE by triparental mating [54]. Triparental mating was used instead of electroporation because there are fewer deletions and rearrangements during mobilization of these large plasmids.

For single-gene cloning, the genomic sequence of *TPR2* and its native promoter (1.0 kb upstream region of the coding sequence) was PCR amplified using the listed primers (Supplementary Table S2). The amplified DNA was cloned into pBlueScriptKSII (-) using *Sma*I, following the Zero Blunt PCR Cloning Kit (ThermoFisher Scientific, Waltham, MA USA) instructions. The cloned DNA was sequenced to confirm gene integrity. This DNA fragment was inserted into pML-BART [55], a plant transformation vector, using *Not*I and *Eco*RV sites. The resultant construct was cloned into ASE by electroporation. The cloning of *SDI2* followed the same procedure. Primers used for *TPR2* and *SDI2* amplification are listed in Supplementary Table S2.

### 4.3. Plant Transformation

Constructs were transformed into wild-type Col-0, Shahdara (Sha), and Tsu-1 plants by floral dipping [56]. Transformed plants were selected by 50 ng/mL kanamycin (pOCA28 vector) or 120 ng/mL BASTA (pMLBART vector). Empty vector constructs were also transformed into wild-type and mutant plants, which were used as negative controls. tpr2 and sdi2 mutants were identified by PCR and gel electrophoresis. Segregation analysis for BASTA resistance was used to identify transformants with a single T-DNA insert. The ratio of plants sensitive and resistant to Basta herbicide (the selectable marker) was evaluated for every T_1_ transformant. At least 100 T_2_ plants were evaluated. If segregation differed from the expected ratio for single gene insertions (75% resistant and 25% sensitive plants), then the T_1_ transformant was not used for further analysis.

### 4.4. Transcript Analysis

RNA was isolated from flowers at anthesis using the RNeasy mini kit (Qiagen, Valencia, CA, USA). Complementary DNA was made using SuperScript III reverse transcriptase (Invitrogen, Grand Island, NY, USA) as recommended by the manufacturer. Quantitative PCR (qPCR) was performed in optical 96-well plates using a StepOnePlus machine (Applied Biosystems, Grand Island, NY, USA). Each 10 µL reaction consisted of GoTaq qPCR Master Mix (Promega, Madison, WI, USA), 2 μL of diluted cDNA, and 0.2 μM each of *TPR2* primers. These primers amplify the 3′ end of the gene. The following thermal profile was used: 95 °C for 2 min, 30 cycles of 95 °C for 15 s, and 60 °C for 1 min. C_T_ and standard curve were extracted using ABI StepOne v2.3 software (Grand Island, NY, USA). *UBQ10* was used as an internal control. Melt curve analyses confirmed that only one product was created from each qPCR.

### 4.5. Carbohydrate Analysis

Prior to seed content quantification, average seed mass was determined for each genotype by weighing three replicates of 100 seeds. For each replicate, the number of seeds was calculated using the total weight and average weight. Carbohydrate content is thus reported per seed, in addition to per milligram of dry weight. For sugar analysis, 25 mg of dry mature seeds (aged at 50% RH for 60 days or longer) were weighed and ground in liquid nitrogen with a pinch of sand. The fine ground powder was transferred to a centrifuge tube. Soluble carbohydrates were extracted from this powder by adding 1 mL of 85% ethanol and 10 mg of charcoal (Sigma, C-4386, St. Louis, MO, USA), which absorbs excess pigments. The sample was then heated at 80 °C for 15 min, followed by centrifugation at 14,000 rpm for 5 min. The supernatant was transferred to a new tube, and ethanol was evaporated overnight at 37 °C. The residue was resuspended in 1 mL ddH_2_O, representing soluble carbohydrate content. For glucose analysis, 20 μL samples and glucose standards were added to a 96-well plate with 100 μL Glucose HK Assay reagent (Sigma, G2393), followed by incubation at 30 °C for 30 min. The production of NADPH was photometrically measured at a wavelength of 340 nm by a BioRad Benchmark Microplate Reader. For fructose levels, 1 unit of PGI enzyme (Sigma P5381) was used for each sample. The increase in absorbance at 340 nm represented fructose content. Sucrose was digested by 10 uL of 10 mg/mL invertase (Sigma I4504), and similarly, the production of NADPH was measured photometrically at a wavelength of 340 nm. The pellet left from ethanol extraction was used for starch quantification. The pellet was solubilized by 0.5 mL of 0.2 M potassium hydroxide and heated at 100 °C for 60 min with periodic inversion. A total of 100 μL 1M acetic acid was used to neutralize the base, and 50 μL of Tris-Cl pH 7.2 was added to buffer the reaction. The starch was initially digested by 100 μL of α-amylase (Sigma, A3430) and incubated at 85 °C for 60 min. Then, 0.5 mL of amyloglucosidase (Sigma, A7905) was added to the tube for complete digestion with heating at 55 °C for 60 min. Debris was pelleted by centrifugation, and the glucose in the supernatant was quantified.

### 4.6. Protein Analysis

For protein quantification, 25 mg of seeds were weighed and ground in liquid nitrogen with a pinch of sand in a mortar with a pestle. The fine ground powder was transferred to a centrifuge tube. Total protein was extracted from dry mature seeds using thiourea/urea lysis buffer and the protocol described in Harder et al. [57]. The thiourea/urea lysis buffer contained 7 M urea (Sigma, U-5378), 2 M thiourea (Sigma, T-8656), 4% (*w*/*v*) CHAPS (Sigma, C-9426), 18 mM Tris-Cl (Thermo Fisher), and 14 mM Tris Base (Thermo Fisher, BP152-10). Then, 0.2% (*v*/*v*) Triton-X-100 (Sigma) and 14 mM dithiothreitol (DTT) (Sigma, D-0632) were applied to further solubilize protein. In addition, 100 μg/mL DNase I (Sigma, DN25) and 100 μg/mL RNaseA (Sigma, R-5125) were used to remove DNA and RNA, respectively. The protease inhibitor cocktail (Research Products International, P51200-1, Prospect, IL, USA) was also added to prevent protein degradation. The protein extract was stirred vigorously and centrifuged for 10 min at 4 °C at 14,000 rpm. The supernatant was collected, and the pellet was resuspended and re-extracted a second time using the same procedure as described above. The combined supernatant corresponded to the total protein extract. Proteins were subjected to SDS-PAGE for visualization. Protein concentration was determined using Bradford Reagent containing 0.01% (*w*/*v*) Coomassie Brilliant Blue G-250, 4.7% (*w*/*v*) ethanol, and 8.5% (*w*/*v*) phosphoric acid. The procedure followed that of Bradford [58]. Bovine serum albumin was used to standardize measurements.

### 4.7. Lipid Analysis

Lipid quantification followed the protocol described by Hara and Radin [59] and Li et al. [60], with a few modifications. Briefly, 100 mg of seeds were weighed and ground in liquid nitrogen with a pinch of sand. A total of 2 mL isopropanol (Sigma, 190764) was added, and the sample was heated at 85 °C for 10 min. Then, 3 mL hexane (Thermo Fisher, 42100) was added to the sample and vortexed for 1 min to mix thoroughly. A total of 2.5 mL of 15% (*wt*/*vol*) sodium sulfate (Thermo Fisher, A19890) was added, and the mixture was incubated at room temperature until visible phase separation. The upper organic phase was collected and transferred to a weighed new tube, and the lower aqueous phase was re-extracted using 2 mL 7/2 (*v*/*v*) hexane/isopropanol. The organic solvent was evaporated under liquid nitrogen steam until the mass became constant. The absolute oil amount was measured by subtracting the tube mass from the total mass.

### 4.8. Data Analysis and Presentation

Data were analyzed using R version 4.42 https://cran.r-project.org/bin/windows/base/ (accessed on 12 December 2024). Data were plotted using a box-whisker plot. In these plots, each box shows the inter-quartile range (IQR; Q1 to Q3) and the median. The whiskers then extend to the outermost points or no more than 1.5 × IQR beyond the box. Individual measurements are shown with dots.

## Data Availability

The data that support the findings will be available in Zenodo at 10.5281/zenodo.15230193.

## Supplementary Materials

The following supporting information can be downloaded at https://www.mdpi.com/article/10.3390/ijms26178310/s1. Reference [61] is cited in Supplementary Materials.

## Abbreviations

The following abbreviations are used in this manuscript:

ABA	Abscisic acid
GA	Gibberellin
GWAS	Genome-wide association study
kb	Kilobase
PCR	Polymerase chain reaction
QTG QTL	Quantitative trait gene Quantitative trait locus
SSQ1	Seed size QTL1
TPR2	Tetratricopeptide Repeat Protein 2

## References

[1] Gnan S., Priest A., Kover P.X. (2014). The Genetic Basis of Natural Variation in Seed Size and Seed Number and Their Trade-Off Using Arabidopsis thaliana MAGIC Lines. Genetics.

[2] Sun X., Shantharaj D., Kang X., Ni M. (2010). Transcriptional and Hormonal Signaling Control of Arabidopsis Seed Development. Curr. Opin. Plant Biol..

[3] Ando A., Kirkbride R.C., Qiao H., Chen Z.J. (2022). Endosperm and Maternal-Specific Expression of *EIN2* in the Endosperm Affects Endosperm Cellularization and Seed Size in *Arabidopsis*. Genetics.

[4] Cheng Z.J., Zhao X.Y., Shao X.X., Wang F., Zhou C., Liu Y.G., Zhang Y., Zhang X.S. (2014). Abscisic Acid Regulates Early Seed Development in *Arabidopsis* by ABI5-Mediated Transcription of *Short Hypocotyl Under Blue1*. Plant Cell.

[5] Gomez M.D., Cored I., Barro-Trastoy D., Sanchez-Matilla J., Tornero P., Perez-Amador M.A. (2023). DELLA Proteins Positively Regulate Seed Size in Arabidopsis. Development.

[6] Hu S., Yang H., Gao H., Yan J., Xie D. (2021). Control of Seed Size by Jasmonate. Sci. China Life Sci..

[7] Jiang W.B., Huang H.Y., Hu Y.W., Zhu S.W., Wang Z.Y., Lin W.H. (2013). Brassinosteroid Regulates Seed Size and Shape in Arabidopsis. Plant Physiol..

[8] Liu H., Luo Q., Tan C., Song J., Zhang T., Men S. (2023). Biosynthesis- and Transport-Mediated Dynamic Auxin Distribution during Seed Development Controls Seed Size in Arabidopsis. Plant J..

[9] Baud S., Dubreucq B., Miquel M., Rochat C., Lepiniec L. (2008). Storage Reserve Accumulation in Arabidopsis: Metabolic and Developmental Control of Seed Filling. Arab. Book.

[10] Li N., Li Y. (2015). Maternal Control of Seed Size in Plants. J. Exp. Bot..

[11] Orozco-Arroyo G., Paolo D., Ezquer I., Colombo L. (2015). Networks Controlling Seed Size in Arabidopsis. Plant Reprod..

[12] Baud S., Boutin J.-P., Miquel M., Lepiniec L., Rochat C. (2002). An Integrated Overview of Seed Development in Arabidopsis thaliana Ecotype WS. Plant Physiol. Biochem..

[13] Liu X., Nakajima K.P., Adhikari P.B., Bradford K.J., Wu X., Zhu K., Kagenishi T., Kurotani K., Ishida T., Nakamura M. (2025). Fertilization-Dependent Phloem End Gate Regulates Seed Size. Curr. Biol..

[14] McLaughlin J.E., Boyer J.S. (2004). Sugar-Responsive Gene Expression, Invertase Activity, and Senescence in Aborting Maize Ovaries at Low Water Potentials. Ann. Bot..

[15] Garcia D., Saingery V., Chambrier P., Mayer U., Jürgens G., Berger F. (2003). Arabidopsis haiku Mutants Reveal New Controls of Seed Size by Endosperm. Plant Physiol..

[16] Jofuku K.D., Omidyar P.K., Gee Z., Okamuro J.K. (2005). Control of Seed Mass and Seed Yield by the Floral Homeotic Gene *APETALA2*. Proc. Natl. Acad. Sci. USA.

[17] Li Y., Zheng L., Corke F., Smith C., Bevan M.W. (2008). Control of Final Seed and Organ Size by the DA1 Gene Family in Arabidopsis thaliana. Genes Dev..

[18] Luo M., Dennis E.S., Berger F., Peacock W.J., Chaudhury A. (2005). MINISEED3 (MINI3), a WRKY Family Gene, and HAIKU2 (IKU2), a Leucine-Rich Repeat Kinase Gene, Are Regulators of Seed Size in Arabidopsis. Proc. Natl. Acad. Sci. USA.

[19] Ren D., Wang X., Yang M., Yang L., He G., Deng X.W. (2018). A New Regulator of Seed Size Control in Arabidopsis Identified by a Genome-Wide Association Study. New Phytol..

[20] Schruff M.C., Spielman M., Tiwari S., Adams S., Fenby N., Scott R.J. (2006). The AUXIN RESPONSE FACTOR2 Gene of Arabidopsis Links Auxin Signalling, Cell Division, and the Size of Seeds and Other Organs. Development.

[21] Garcia D., Fitz Gerald J.N., Berger F. (2005). Maternal Control of Integument Cell Elongation and Zygotic Control of Endosperm Growth Are Coordinated to Determine Seed Size in Arabidopsis. Plant Cell.

[22] Guo J., Fan J., Hauser B.A., Rhee S.Y. (2016). Target Enrichment Improves Mapping of Complex Traits by Deep Sequencing. G3 Genes Genomes Genet..

[23] Herridge R.P., Day R.C., Baldwin S., Macknight R.C. (2011). Rapid Analysis of Seed Size in Arabidopsis for Mutant and QTL Discovery. Plant Methods.

[24] Wang D., Sun W., Yuan Z., Sun Q., Fan K., Zhang C., Yu S. (2021). Identification of a Novel QTL and Candidate Gene Associated with Grain Size Using Chromosome Segment Substitution Lines in Rice. Sci. Rep..

[25] Zhang S., Hu X., Miao H., Chu Y., Cui F., Yang W., Wang C., Shen Y., Xu T., Zhao L. (2019). QTL Identification for Seed Weight and Size Based on a High-Density SLAF-Seq Genetic Map in Peanut (*Arachis hypogaea* L.). BMC Plant Biol..

[26] Alonso-Blanco C., Vries H.B.-D., Hanhart C.J., Koornneef M. (1999). Natural Allelic Variation at Seed Size Loci in Relation to Other Life History Traits of *Arabidopsis thaliana*. Proc. Natl. Acad. Sci. USA.

[27] Liang S., Duan Z., He X., Yang X., Yuan Y., Liang Q., Pan Y., Zhou G., Zhang M., Liu S. (2024). Natural Variation in *GmSW17* Controls Seed Size in Soybean. Nat. Commun..

[28] Nguyen C.X., Paddock K.J., Zhang Z., Stacey M.G. (2020). GmKIX8-1 Regulates Organ Size in Soybean and Is the Causative Gene for the Major Seed Weight QTL *qSw17-1*. New Phytol..

[29] Lin F., Fan J., Rhee S.Y. (2019). QTG-Finder: A Machine-Learning Based Algorithm to Prioritize Causal Genes of Quantitative Trait Loci in Arabidopsis and Rice. G3 Genes Genomes Genet..

[30] Kvilekval K., Fedorov D., Obara B., Singh A., Manjunath B.S. (2009). Bisque: A Platform for Bioimage Analysis and Management. Bioinformatics.

[31] Winter D., Vinegar B., Nahal H., Ammar R., Wilson G.V., Provart N.J. (2007). An “Electronic Fluorescent Pictograph” Browser for Exploring and Analyzing Large-Scale Biological Data Sets. PLoS ONE.

[32] Weigel D., Mott R. (2009). The 1001 Genomes Project for Arabidopsis thaliana. Genome Biol..

[33] Parker M.T., Soanes B.K., Kusakina J., Larrieu A., Knop K., Joy N., Breidenbach F., Sherwood A.V., Barton G.J., Fica S.M. (2022). m6A Modification of U6 snRNA Modulates Usage of Two Major Classes of Pre-mRNA 5′ Splice Site. eLife.

[34] Prasad B.D., Goel S., Krishna P. (2010). In Silico Identification of Carboxylate Clamp Type Tetratricopeptide Repeat Proteins in Arabidopsis and Rice as Putative Co-Chaperones of HSP90/HSP70. PLoS ONE.

[35] Angeles-Núñez J.G., Tiessen A. (2011). Mutation of the Transcription Factor LEAFY COTYLEDON2 Alters the Chemical Composition of Arabidopsis Seeds, Decreasing Oil and Protein Content, While Maintaining High Levels of Starch and Sucrose in Mature Seeds. J. Plant Physiol..

[36] Durand M., Mainson D., Porcheron B., Maurousset L., Lemoine R., Pourtau N. (2018). Carbon Source–Sink Relationship in Arabidopsis thaliana: The Role of Sucrose Transporters. Planta.

[37] Weber H., Borisjuk L., Wobus U. (1997). Sugar Import and Metabolism during Seed Development. Trends Plant Sci..

[38] Angeles-Núñez J.G., Tiessen A. (2010). Arabidopsis Sucrose Synthase 2 and 3 Modulate Metabolic Homeostasis and Direct Carbon Towards Starch Synthesis in Developing Seeds. Planta.

[39] Rosado A., Schapire A.L., Bressan R.A., Harfouche A.L., Hasegawa P.M., Valpuesta V., Botella M.A. (2006). The Arabidopsis Tetratricopeptide Repeat-Containing Protein TTL1 Is Required for Osmotic Stress Responses and Abscisic Acid Sensitivity. Plant Physiol..

[40] Yang C., Li X., Chen S., Liu C., Yang L., Li K., Liao J., Zheng X., Li H., Li Y. (2023). ABI5–FLZ13 Module Transcriptionally Represses Growth-Related Genes to Delay Seed Germination in Response to ABA. Plant Commun..

[41] Lin Z., Ho C.W., Grierson D. (2009). AtTRP1 Encodes a Novel TPR Protein That Interacts with the Ethylene Receptor ERS1 and Modulates Development in Arabidopsis. J. Exp. Bot..

[42] Lin Z., Arciga-Reyes L., Zhong S., Alexander L., Hackett R., Wilson I., Grierson D. (2008). SlTPR1, a Tomato Tetratricopeptide Repeat Protein, Interacts with the Ethylene Receptors NR and LeETR1, Modulating Ethylene and Auxin Responses and Development. J. Exp. Bot..

[43] Filardo F., Robertson M., Singh D.P., Parish R.W., Swain S.M. (2008). Functional Analysis of HvSPY, a Negative Regulator of GA Response, in Barley Aleurone Cells and Arabidopsis. Planta.

[44] Jacobsen S.E., Olszewski N.E. (1993). Mutations at the SPINDLY Locus of Arabidopsis Alter Gibberellin Signal Transduction. Plant Cell.

[45] Tseng T.-S., Swain S.M., Olszewski N.E. (2001). Ectopic Expression of the Tetratricopeptide Repeat Domain of SPINDLY Causes Defects in Gibberellin Response. Plant Physiol..

[46] Fenn M.A., Giovannoni J.J. (2020). Phytohormones in Fruit Development and Maturation. Plant J..

[47] Hirano T., Kinoshita N., Morikawa K., Yanagida M. (1990). Snap Helix with Knob and Hole: Essential Repeats in S. pombe Nuclear Protein nuc2 ^+^. Cell.

[48] Sikorski R.S., Boguski M.S., Goebl M., Hieter P. (1990). A Repeating Amino Acid Motif in CDC23 Defines a Family of Proteins and a New Relationship among Genes Required for Mitosis and RNA Synthesis. Cell.

[49] Debeaujon I., Lepiniec L., Pourcel L., Routaboul J. (2007). Seed Coat Development and Dormancy. Annual Plant Reviews.

[50] Braun D.M. (2022). Phloem Loading and Unloading of Sucrose: What a Long, Strange Trip from Source to Sink. Annu. Rev. Plant Biol..

[51] Park S.O., Hwang S., Hauser B.A. (2004). The Phenotype of Arabidopsis Ovule Mutants Mimics the Morphology of Primitive Seed Plants. Proc. Biol. Sci..

[52] Weis J.H., Quertermous T. (1987). Size Fractionation Using Sucrose Gradients. Curr. Protoc. Mol. Biol..

[53] Olszewski N.E., Martin F.B., Ausubel F.M. (1988). Specialized Binary Vector for Plant Transformation: Expression of the Arabidopsis thaliana AHAS Gene in Nicotiana tabacum. Nucleic Acids Res..

[54] Fraley R.T., Rogers S.G., Horsch R.B., Eichholtz D.A., Flick J.S., Fink C.L., Hoffmann N.L., Sanders P.R. (1985). The SEV System: A New Disarmed Ti Plasmid Vector System for Plant Transformation. Nat. Biotechnol..

[55] Gleave A.P. (1992). A Versatile Binary Vector System with a T-DNA Organisational Structure Conducive to Efficient Integration of Cloned DNA into the Plant Genome. Plant Mol. Biol..

[56] Clough S.J., Bent A.F. (1998). Floral Dip: A Simplified Method for Agrobacterium-Mediated Transformation of Arabidopsis thaliana. Plant J..

[57] Harder A., Wildgruber R., Nawrocki A., Fey S.J., Larsen P.M., Görg A. (1999). Comparison of Yeast Cell Protein Solubilization Procedures for Two-Dimensional Electrophoresis. Electrophoresis.

[58] Bradford M. (1976). A Rapid and Sensitive Method for the Quantitation of Microgram Quantities of Protein Utilizing the Principle of Protein–Dye Binding. Anal. Biochem..

[59] Hara A., Radin N.S. (1978). Lipid Extraction of Tissues with a Low-Toxicity Solvent. Anal. Biochem..

[60] Li Y., Beisson F., Pollard M., Ohlrogge J. (2006). Oil Content of Arabidopsis Seeds: The Influence of Seed Anatomy, Light and Plant-to-Plant Variation. Phytochemistry.

[61] Cheng C., Krishnakumar V., Chan A.P., Thibaud-Nissen F., Schobel S., Town C.D. (2017). Araport11: A Complete Reannotation of the Arabidopsis thaliana Reference Genome. Plant J..

