# Supplementary material for: Tetratricopeptide Repeat 2 Is a Quantitative Trait Locus That Controls Seed Size
Source: Int J Mol Sci. 2025 Aug 27;26(17):8310. doi: 10.3390/ijms26178310 (PMC12428041; doi:10.3390/ijms26178310)
Supplement: Supplementary file 1 [file ijms-26-08310-s001.zip › ijms-3725168-supplementary.pdf]

Supplemental Table S1. Sucrose levels in Sha-1, Col-0, and Tsu-1 accessions

	sucrose concentration (μg/mg)
Col-0	20.4±0.7
Sha-1	19.4±0.4
Tsu-1	18.3±0.4

Supplemental Table S2. Primers used in this study

Primer ID	Sequence (from 5' to 3')	Purpose
TPR2-LP	TGCGTGGTCCTTTAACAAAAG	Genotyping
TPR2-RP	TTTGAAGCATTGAAACCCATC	Genotyping
SDI2-LP	CGACACCCGAAAAAGCTTA	Genotyping
SDI2-RP	AGCTGAAGAAGCCATTGACG	Genotyping
LBb1.3	ATTTTGCCGATTTCGGAAC	Genotyping
TPR2-2L	TGTGGGATACAATGGAGGAGGAAC	Cloning
TPR2-2R	CGACAACCAACAACAAAAAGAAGC	Cloning
SDI2-2L	CCAAACTAATGATCCGAGTCACCC	Cloning
SDI2-2R	GAATGAGATCATTTGACATTGCGG	Cloning

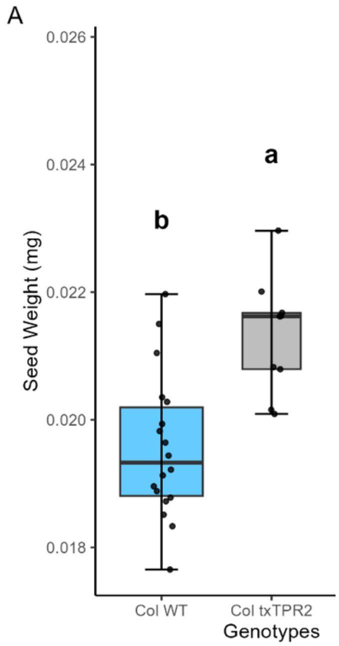

Supplementary Figure S1. Seed mass of cosmid transformants in Col-0 genetic background ($N \geq 8$). A) Seed mass from Col-0 plants transformed with a (tx) *TPR2*-containing cosmid. The *TPR2* transgene increased seed mass by 11.8% in the Col-0 background. Significant differences were calculated using ANOVA and Tukey HSD and denoted by letters.

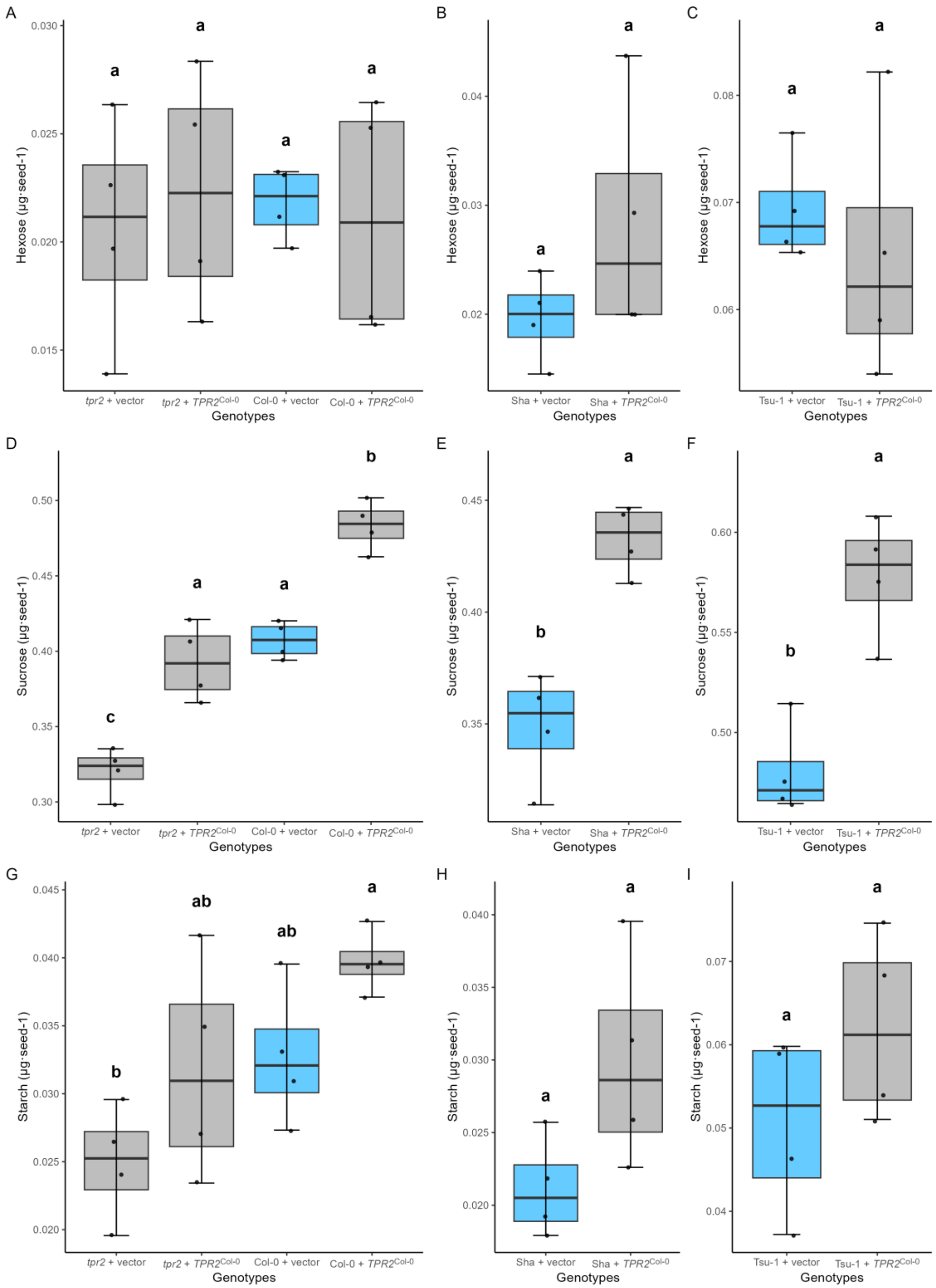

Supplemental Figure S2. Carbohydrate amount per seed in different genotypes (n = 4 biological replicates). A-C, Hexose (glucose + fructose) in Col-0, Sha, and Tsu-1 accessions. D-F, Sucrose in Col-0, Sha and Tsu-1. G-I Starch in Col-0, Sha, and Tsu-1. Data were the mean of four independent measurements from four individual plants. Significant differences were calculated using ANOVA and Tukey HSD and denoted by letters.

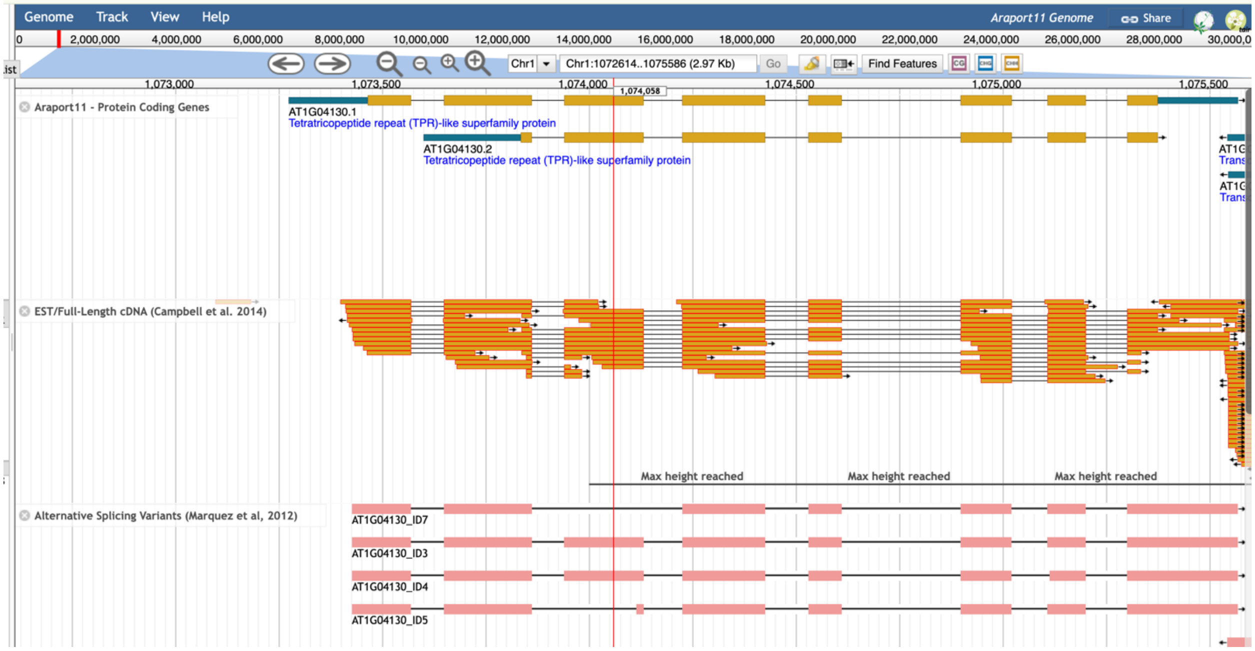

Supplemental Figure S3. The transcripts produced by *TPR2* are annotated by Araport11 genome viewer [61]. In addition to two difference transcription start sites, this TPR2 shows four splice variants. The splice variants with some or all of exon3 missing (ID7 and ID5), will lack most of the TPR domain, which is involved in binding other proteins. The red line runs through the third exon. The At1g04130_ID4 variant uses an alternate splice acceptor site for intron 6, resulting in multiple downstream stop codons.

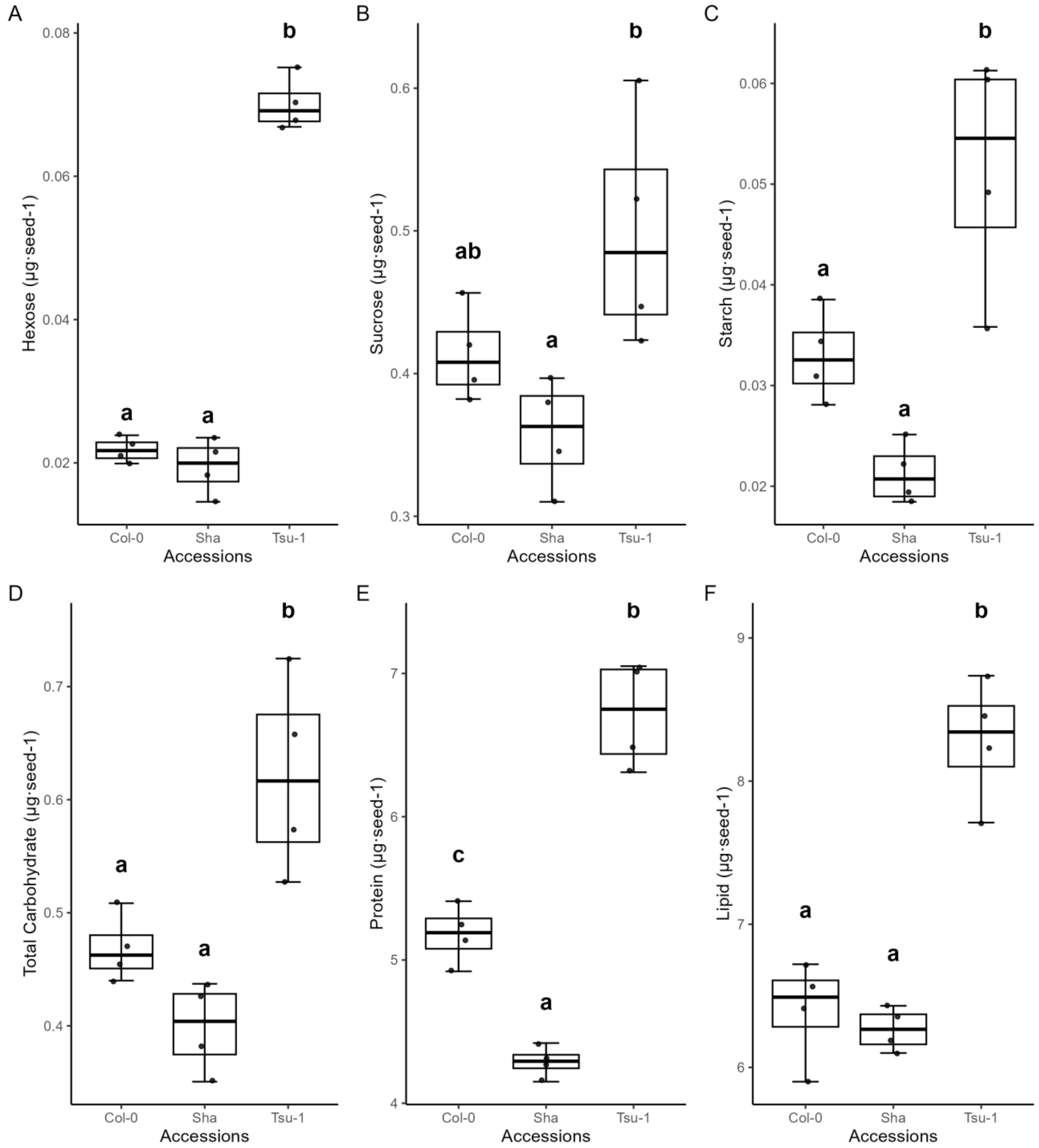

Supplemental Figure S4. Seed metabolite levels in different wild-type *Arabidopsis thaliana* accessions are shown on a per seed basis (n = 4 biological replicates). A-C, Amount of hexose (glucose + fructose), sucrose, and starch in single seeds in Sha, Col-0 and Tsu-1. D-F. Total carbohydrate, protein, and oil in individual Col-0, Sha, and Tsu-1 seeds. Data are the mean of four independent measurements from individual plants. P values of different comparisons were shown above the boxes.
